# Babycare Assistance Needs of Parents With Physical Disabilities: An Observational Study

**DOI:** 10.1177/15394492231172935

**Published:** 2023-05-16

**Authors:** Evelina Pituch, Tiffanie Cormier, Véronique Gilbert, Carolina Bottari

**Affiliations:** 1 Université de Montréal, Québec, Canada; 2 CIUSSS du Centre-Sud-de-l’Île-de-Montréal, Québec, Canada

**Keywords:** Activities of Daily Living, assessment, parenting, cognition, occupational therapy

## Abstract

The assistance needs of parents with physical disabilities have been widely underexamined. This qualitative observational study described the assistance needs of parents with physical disabilities during the performance of in-home babycare activities. Thirty-one parents were assessed by trained occupational therapists using the Activities of Daily Living (ADL) Profile adapted for use with parents, an ecological performance-based assessment that considers executive functioning. Descriptive statistics of participants’ demographics and parents’ independence scores in babycare activities were calculated, and a qualitative content analysis of parents’ assistance needs was performed using video recordings. At least one-fourth of parents experienced difficulties in all babycare activities, either affecting activity performance or requiring verbal or physical assistance. Assistance needs were also observed in all activity-related operations of the ADL Profile. It is necessary to develop specialized clinical services to address assistance needs and promote safe and easy parenting in parents with physical disabilities.

## Introduction

Although 6.2% of parents of minor children have physical disabilities (Kaye, 2012), the assistance needs of these parents have been widely underexamined and underreported. Clinical services that meet these parents’ needs and right to parent (National Council on Disability, 2012) remain scarce, with insufficient supports and potential risks to babycare (Wint et al., 2016).

Parenting is an occupation with several dynamic competing demands that requires not only a broad array of physical skills, but more importantly, the input of high-level cognitive functions known as executive functions (EFs) (Cramm et al., 2016). According to the American Occupational Therapy Association (2020), parenting or “child rearing” is an instrumental activity of daily living defined as “providing care and supervision to support the developmental and physiological needs of a child” (p. 31), whereas EF are described as higher level cognitive functions (pp. 51–52). Although Cramm et al. (2013) found conceptual variance of this construct, EF are broadly understood as the ability to initiate, plan, organize, monitor, prioritize, and utilize daily living information in complex goal-directed behaviors. Although physical impairments can often be compensated with adaptive equipment (Jacob et al., 2017), the same cannot be said for EF impairments which are known to be present in nearly all brain-related diagnoses (Wolf & Baum, 2011). Hence, it is urgent that we examine parental babycare assistance needs in consideration of the possible impact of EF in parents with physical disabilities.

Previous studies of parents with physical disabilities have highlighted the importance of EF, or more specifically planning ahead or “preplanning” as a key parental strategy to facilitate family functioning and avoid unforeseen events or emergencies in terms of scheduling, sharing chores, and outings in child-friendly accessible places (Bergeron et al., 2012; McKeever et al., 2003). However, a recent scoping review of the needs of parents with physical disabilities highlighted the limited knowledge surrounding the specific activity-related challenges that arise in child care, particularly in the presence of cognitive impairments (Pituch et al., 2020).

Dynamic performance-based assessments of everyday activities have the potential of facilitating a better understanding of the impact of EF impairments on complex daily activities, such as parenting (Bottari et al., 2009; Cramm et al., 2016; Ylvisaker et al., 2003), if the assessments are developed for this specific goal (Major et al., 2018). However, previous authors have acknowledged the inherent challenges of measuring parenting, partly explained by underlying contextual and cultural factors and the analysis of multifaceted behaviors (Lindhiem et al., 2019). In clinical settings, the challenges associated with observation-based assessments may limit their use, despite being considered a gold standard for parenting assessments (Hawes & Dadds, 2006). More specifically, in occupational therapy, a discipline in which observational skills are foundational (Stigen et al., 2023), the current lack of formal observation-based methods validated for parents with physical disabilities (Pastor-Bédard et al., 2023) further limits therapists’ ability to assess parents’ babycare assistance needs. Moreover, while it is often advanced that parents with physical disabilities have support needs (National Council on Disability, 2012), it is unclear what babycare activities and activity-related components are most challenging and to what extent. Understanding activity breakdowns during assessments is important to elicit causal factors (Ylvisaker et al., 2003) and facilitate future parental activity-related success. Thus, this study’s objective was to describe the babycare assistance needs of parents with physical disabilities, both mothers and fathers. To do so, we used the *Activities of Daily Living (ADL) Profile adapted for use with parents* (Major et al., 2018), an occupational therapy assessment that considers EF and was specifically developed to assess babycare independence and related assistance needs. Hence, our research questions were:

Research Question 1 (RQ1): How do parents with disabilities perform babycare activities?
Research Question 2 (RQ2): What assistance do parents need when performing common babycare activities and where do activity-related breakdowns occur?

Investigating such questions may help develop better clinical services for parents with physical disabilities.

## Method

This study was done in collaboration with the *Parents Plus Clinic*, a university affiliated parenting clinic that offers specialized occupational therapy rehabilitation services to parents with a physical disability, including in-home and/or consultation assessments and interventions, and off-market subsidized adaptive parenting equipment in Montreal, Canada (Clinique Parents Plus - CIUSSS CSMTL, 2022). Clinical occupational therapists (OTs) identified potential participants from their regular caseloads and assessments were completed in dyads with an occupational therapy researcher. Below, the reporting of our research complies with the COnsolidated criteria for REporting Qualitative research checklist (Tong et al., 2007).

### Design

A qualitative observational descriptive study (Sandelowski, 2010) following a constructivist paradigm (Guba & Lincoln, 1998) was undertaken using continuous video recordings of parents’ babycare activities in their home environment. Video methods are used across disciplines to study complex occupations (Pierce, 2005). This study was preceded by a pilot study published elsewhere (Major et al., 2018). This study was embedded within an integrated knowledge transfer approach (Andrews et al., 2015) in which occupational therapy researchers and clinical OTs co-assessed parents and mutually shared their related expertise during the assessments and analyses. This approach facilitated the complex observational assessment of parenting and uptake of the assessment in a clinical setting. Assessments results were used clinically to guide treatment interventions that were outside the scope of this study.

### Participants

Eligible participants were adult parents of babies up to 2 years old receiving ongoing services at the parenting clinic. Parents had to have diagnosed, self-reported, or suspected cognitive impairments. To select information-rich cases, parent participants were recruited using purposeful sampling. Sampling saturation was determined by maximum variation sampling (Patton, 2015) in terms of parent diagnoses, living situation, parenting experience, number of children, child(ren) age, mobility and parenting equipment used, and child welfare involvement. No exclusion criterion was applied. The study was approved by the *Research Ethics Board in rehabilitation and in physical disability of the CIUSSS du Centre-Sud-de-l’Île-de-Montréal* (CRIR-1015-1114) and all participants provided written informed consent. Participants could withdraw at any time from the study, but none did.

### Measure

The *ADL Profile adapted for use with parents* (Major et al., 2018), used in our pilot study and in this broader study, is an alternative version of the *ADL Profile*. Initially developed for adults who sustained a traumatic brain injury, the *ADL Profile* is an ecological performance-based measure of independence, and related assistance needs, in everyday activities. Direct observations of both simple and complex activities, and familiar and novel activities are integral to the assessment (Bottari et al., 2020; Dutil et al., 2005). With its strong underpinnings in the EF literature, independence measured by the *ADL Profile* is defined as the ability to formulate a goal, plan, carry out a task, and verify goal attainment (Lezak, 1982; Luria, 1973), which are also the four components of EF assessed for each observed activity, as defined in Table 1. By using a dynamic assessment approach (Ylvisaker et al., 2003) with minimally structured instructions, it aims to solicit EF, particularly goal formulation and planning, document optimal abilities, and guide future interventions. The *ADL Profile* has established test–retest reliability (Dutil et al., 2017), structural validity, internal consistency, and clinical applicability (Bottari et al., 2020).

**Table 1.** Definitions of EF-Based Operations (Dutil et al., 2005).

EF-based operations	Operational definitions
Formulate a goal	Refers to expressing (verbally or internally) a solution to satisfy a need or solve a problematic situation (i.e., initial goal).
Plan	Refers to thinking before acting about the starting conditions, identifying alternatives, choosing the most appropriate alternative, developing a general strategic/tactical plan of action (sequence of actions or steps), and gathering and preparing materials for the task.
Carry out a task	Refers to initiating the plan of action, continuing to carry out the plan of action (including continuous monitoring/checking of the execution compared to the initial goal) while adjusting to perceived errors and new or unforeseen situations, perceiving errors in planning (e.g., in estimating time, space) and execution (e.g., in handling, in tool selection), and modifying the execution according to perceived errors and unforeseen situations.
Verify attainment of goal	Refers to identifying the achievement of the initial goal (comparing the results obtained with the initial goal), accepting or rejecting the results, completing the task, or restarting the process when the result is rejected (i.e. the initial goal not being achieved).

*Source.* Dutil et al. (2005).

*Note.* EF = executive functioning.

The *ADL Profile adapted for use with parents* consists of eight babycare activities, namely “bathing the baby,” “dressing” (including diaper changing), “preparing meal(s)” (including bottle preparations), “feeding,” “going outside,” “putting baby to sleep,” “playing,” and “obtaining babycare information,” though parents are not told this as they are invited to decide the activities they need to carry out with their baby as though they were alone. This is an important feature of the *ADL Profile* and it distinguishes it from other standardized assessments that are more prescriptive or manualized in nature. In the *ADL Profile*, control of the assessment is largely transferred from the assessor to the parent. More specifically, initial instructions of the *ADL Profile adapted for use with parents*, as adapted from the *ADL Profile*, are simply the following: “*I would invite you to do babycare as you would normally do it*.” Parents were further informed that any required assistance would be provided by the OT who would be present to ensure the safety of the parent and the baby. Then, salient observations of assistance needs (e.g., type, frequency, importance) are recorded and qualitatively analyzed to permit scoring. For scoring, observed assistance needs are translated into a four-level ordinal scale as the original version was used: 3—independence without difficulty; 2—independence with difficulty; 1—requires verbal, physical, or verbal and physical assistance; 0—dependence. Each activity-related operation (goal formulation, planning, carrying out, verifying goal attainment) was scored using this four-level ordinal scale. The overall activity score is the lowest of the four activity-related operation scores with task scores reflecting the weakest links in performance, or assistance needs. Face validity was established through exchanges between the researchers and OTs specialized in parenting to ensure that the assessment reflected clinical practice needs.

### Assessors

The assessors were nine female OTs and one female professional master’s student in OT, including six clinical OTs (range: 10–25 years of experience at first assessment), all with experience with parents with disabilities. Each clinical OT participated in an average of 5.2 assessments (range: 1–9 assessments). Prior to data collection, all but two engaged in a three-day credited continuing education on the *ADL Profile*. The remaining OT and the OT student received individualized training by the first (EP) or last (CB) author. In addition, all OTs had access to an unpublished 100-page assessment manual, collaboratively created, providing background context, assessment and scoring guidelines, and note-taking material. At all times during the study, OTs had easy access to the first (EP) and last authors (CB), who is also one of the *ADL Profile* co-authors (Dutil et al., 2005), to address any assessment-related issues.

### Data Collection

Data collection spanned approximately 6 years between February 2015 and October 2021 as the new assessment was progressively introduced into clinical practice and OTs obtained necessary training. Assessments were conducted in either French or English, following the parent’s preference. Each parenting assessment was administered once during approximately 3 continuous hours. Parents’ sociodemographic data were collected through clinical records.

Dyads of trained OTs, specifically one researcher and one clinician, conducted announced in-home parenting assessments recorded in-person using a hand-held video camera. In the case of geographical barriers, the dyads engaged in remote assessments with a team of collaborating on-site OTs from regional clinics involved in the participants’ care. For the latter, the trained OTs administered the assessment *per se* and the onsite OTs provided required assistance and ensured parent and baby safety, with wi-fi connected equipment (e.g., smartphone, tablet) sharing live footage which was then locally recorded by the dyads using a secured and password-protected desktop computer. Video recording and note-taking were performed as discretely as possible to avoid biasing parent-baby interactions or affecting the family’s intimacy.

To observe information-rich babycare activities, the assessment was scheduled in consideration of each family’s schedule and baby routines. The assessment itself was initiated with the start of babycare, after providing instructions and answering all questions. To ensure that parents remained in control of their baby’s care to the full extent of their ability to do so, specific attention was given not to unnecessarily distract, prompt, cue, or assist parents during the assessments. When parents requested assistance, assessors first encouraged parents to problem-solve on their own using a think-aloud approach, inciting parents to explore alternative solutions they could identify on their own. When a situation was judged by the assessor as having an overtly high-risk situation for the parent or the baby, the assessor gave specific and immediate actions or fully took control of the activity at hand. If present at home, non-participants (e.g., family members) were asked not to intervene during the assessment. Near to the end of the assessment, context permitting, the examiner proposed other babycare activities (e.g., bathing, going outside, obtaining babycare information).

Finally, prior to this study and to compensate for physical impairments, nearly all parents received in-home adaptive parenting equipment for which they received training prior to the assessment, as suggested by previous authors in the field (National Council on Disability, 2012). Adaptive parenting equipment was provided following clinical occupational therapy assessments at the *Parents Plus Clinic*, which were outside the scope of the current study.

### Data Analysis

Data analysis consisted of descriptive statistics and qualitative content analysis of salient assistance needs with three iterative rounds of coding using pilot-tested Microsoft Excel© activity grids. First, following each assessment, qualitative observations of babycare activities and corresponding independence scores were collaboratively discussed by the OT assessor dyads until a mutual analysis of key observations was attained. To avoid incorrect recollections, video excerpts were reviewed when necessary. Any scoring disagreements between co-assessors were resolved through extended observation-based discussions. If necessary, the last author (CB) helped reach a consensus in a consecutive meeting based on select reported observations. Second, although intra-rater reliability was not documented, the first author (EP) performed a cross-participant deductive coding of all operation and activity scores using the *ADL Profile*’s operational definitions (Table 1) and key observations. A mixed (deductive and inductive) video content analysis (Nassauer & Legewie, 2021) of parents’ salient behaviors and assistance needs followed, with in-depth analyses of all recordings and observation notes performed by EP (e.g., actions, interactions, contexts). Inductive coding was necessary to capture emerging categories of behaviors or assistance needs not featured in the original *ADL Profile* assessment but having an impact on scoring. Third, despite not establishing inter-rater reliability, to ensure the trustworthiness of data analysis (Tracy, 2010), the second author (TC), not involved in the co-assessments, independently analyzed a third of the recordings and scores and assistance needs were compared until a final consensus was reached.

## Findings

### Participants’ Demographics and Parenting Data

A total of 31 biological parent-baby dyads participated, namely, 22 mothers (71%), 9 fathers (29%), and their 29 babies (14 girls, 15 boys), for whom demographics are presented in Table 2. Of these, 22 (71%) participants were first-time parents, including 16 new mothers (73%) and six new fathers (27%). Our sample was diverse in terms of parent diagnoses, living situation, parenting experience (range: 1 month-11 years), number of children, child(ren) age, and child welfare involvement. Twenty-six (84%) parents also had diagnosed or self-reported one or concurrent cognitive disabilities such as attention/concentration difficulties (*n* = 14), memory difficulties (*n* = 12), learning difficulties (*n* = 7), inhibition difficulties (*n* = 5), EF impairments (*n* = 3), an intellectual disability (*n* = 3), organization difficulties (*n* = 1), or dyscalculia (*n* = 1). The remaining 16% had suspected, but not diagnosed cognitive impairments. Our sample also varied in terms of mobility and parenting equipment used. Participants lived in 9 out of the 16 administrative regions in the Quebec province. Four parents were related (two couples) and assessed individually. One parent participated with the support of sign language interpreters.

**Table 2.** Parents’ and Children’s Demographic Data at the Time of Assessment (n = 31).

Parent characteristics	*M* (*SD*) or *n*
Age (years)	33.3 (6.6)
Range	19.1–48.2
Age at last childbirth (years), *n* = 31	32.9 (6.5)
Range	17.9–48.1
Main parent physical disability	
Brain injuries	16
Cerebral palsy	7
Stroke (including perinatal)	4
Traumatic brain injury	3
Cerebral hypoxia or anoxia (including during delivery)	2
Neuromuscular disorders	10
Multiple sclerosis^a^	3
Ataxia (cerebellar, spinocerebellar, autosomal recessive spastic ataxia of Charlevoix-Saguenay)	3
Myotonic dystrophy type 1 (Steinert’s disease)	2
Charcot-Marie-Tooth disease	1
Huntington’s disease	1
Other	5
Post-polio syndrome	2
Spina bifida	1
Epilepsy^a^	1
Functional neurological disorder	1
Parental disability status	
Prior to birth of youngest child	30
During child delivery	1
Ethnicity	
Caucasian	23
Black	7
Hispanic	1
Civil status	
In couples	21
Single	10
Living situation^b^	
With partner	20
With other family member(s) or roommate(s)	14
Alone	5
Mobility aid used by parent^c^	
Manual wheelchair	10
Power wheelchair	1
Walker	1
Parent has legal custody	
Yes	25
No	6
Number of children (*n* total = 44)	
1	22
2	5
3	2
4	2
Children characteristics	*M* (*SD*) or *n*
Youngest child’s chronological age (months)	6.2 (4.6)
Range	1–15.2
Child disability or medical condition that needs to be considered in babycare	
Yes	4
No	27
Child protection services involved	
Yes	8
No	21

a Some parents presented multiple diagnoses, including cerebral palsy (*n* = 2), morbid obesity (*n* = 1), hearing impairments (*n* = 3), visual impairments (*n* = 4), and autism under investigation (*n* = 1). ^b^ This category is not mutually exclusive, as some parents lived with partners and other family members or roommates. ^c^ This category is not mutually exclusive, nor exhaustive. Some parents used more than one mobility equipment during the assessment, while others also used other mobility equipment, outside the scope of this assessment (e.g., for outdoor longer distances).

This study was conducted in-person in participants’ homes (*n* = 29), except for two participants who were assessed in their relatives’ homes, a familiar setting. For eight parents (26%), remote assessments were performed using secured videoconferencing software. Data collection was diverse in terms of assessors (12 dyads) and year (about 4.4 assessments/year), though one of only two OT researchers participated in each assessment.

**Figure 1.**
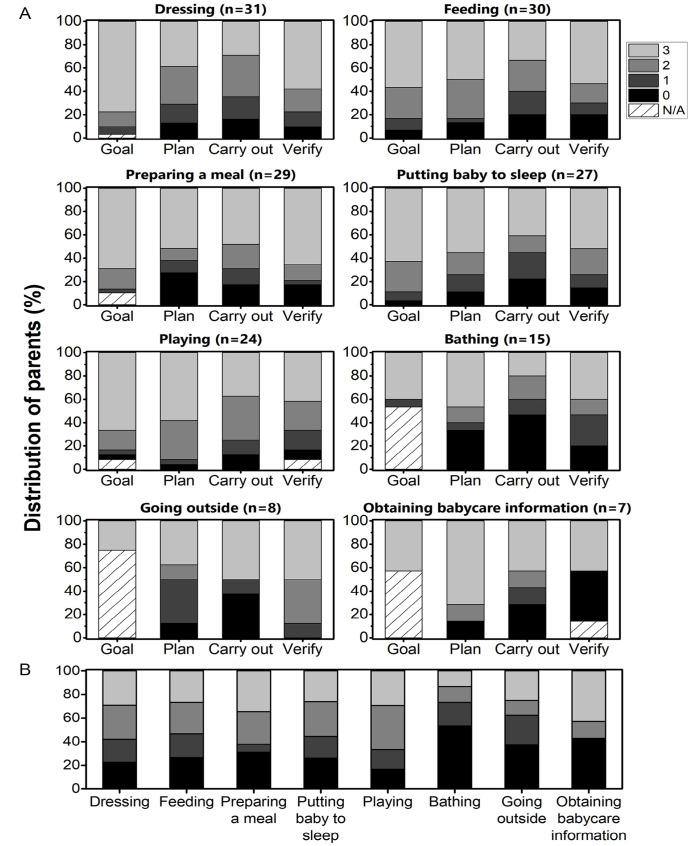
Distribution of Parents’ Babycare Independence for Each Operation (A) and for Total Activity Scores (B) of the Eight Parenting Activities Assessed. *Note.* Scores: 3—independence without difficulty, 2—independence with difficulty, 1—requires verbal and/or physical assistance, 0—dependence. N/A signifies that the activity was either requested by the assessors (i.e., formulate a goal) or that the corresponding operation(s) could not be observed and therefore scored during the allocated assessment period. Six mothers breastfed during the feeding activity.

Parents accomplished on average 5.5 parenting activities (range: 4–8 parenting activities). When considering our total sample, each parenting activity was performed on average 21.4 times (range: 7–31 times), although “going outside” and “obtaining babycare information” was accomplished by only a fifth of our sample. Figure 1 shows the distribution of parents’ babycare performance per operation and activity. Of note, all four independence levels were used to score the parenting activities of our sample, suggesting that parents varied in terms of need for babycare assistance. When considering the total activity score, “bathing” was the activity with the lowest scores (53.3% were dependent), particularly for the planning and carrying out operations, whereas “obtaining babycare information” was the activity with both the highest number of parents who were independent and the highest number of parents who were dependent, particularly during the planning and verifying goal attainment operations. At least one fourth of our sample had difficulties or required assistance (verbal and/or physical) at some point during the six other parenting activities assessed. Finally, Table 3 highlights the 13 babycare adaptive pieces of equipment that were used by parents (81%) during their parenting activities, with wheelchair-accessible cribs and breastfeeding cushions being the most widely used.

**Table 3.** Parents Plus Babycare Adaptive Equipment Used by Parents During Assessments.

Babycare adaptive equipment	Equipment description	Related babycare activities during which the equipment was used	Sample size
Ottawa bed	WC accessible crib w/ lateral opening and locking mechanisms and security locks	Putting baby to sleep Dressing Playing w/ baby Feeding	13
Traveler cushion	Adapted breastfeeding cushion for WC travels w/ adjustable parent belt and/or child safety strap or harness	Feeding	10
Angel’s pad	Changing pad w/ safety harness w/ adjustable straps	Dressing	5
Adapted baby bassinet	Baby bassinet attached to an adjustable rolling base w/ brakes	Putting baby to sleep	5
Lilou vest	Child vest w/ textile handles to facilitate parent’s grip during transfers	Dressing Putting baby to sleep Feeding	4
Adjustable sideboard	Child booster seat attached to an adjustable rolling base w/ brakes	Feeding Preparing meal(s)^a^	4
Otter bathtub	Baby bathtub attached to an adjustable rolling base w/ brakes	Bathing	4
Mobile cachou	Child booster seat attached to a rollator	Feeding Preparing meal(s)^a^	3
Cigogne sling	Small soft mattress w/ child harness and straps held by the parent during baby transfers	Putting baby to sleep	2
Ankle brace	Soft and adjustable wrap-around ankle fabric strap suited for children	Dressing	2
Beveled cushion	Angled cushion w/ strap to be attached around parent’s waist	Going outside Feeding	2
Kangaroo seat	Adapted child booster seat w/ child five-point harness and two parent straps to hold child and equipment onto parent	Going outside	1
Double belt	Child waist belt attached to parent’s WC belt	Going outside	1

*Note.* Some parents had access to other adaptive parenting equipment at home, but did not use it during the assessment. WC = wheelchair; w/ = with.

a This equipment was used to watch the child during meal preparation. Additional information regarding the Parents Plus adaptive equipment is available here (Clinique Parents Plus - CIUSSS CSMTL, 2022).

### Qualitative Content Analysis

Finally, Table 4 presents a total of 36 emerging categories of salient parent behaviors or needs, classified according to the babycare independence levels and activity operations of the *ADL Profile adapted for use with parents*. Each independence level has between 8 and 10 categories illustrative of salient babycare behaviors or assistance needs. In addition, illustrated in Table 4 are the parenting activities in which the latter were observed.

**Table 4.** Salient Observations Regarding Parental Strengths, Difficulties, and Assistance Needs Classified Using the ADL Profile Adapted for Use With Parents.

Babycare independence score	Salient observations		Babycare activities							
			Bathing	Dressing	Preparing meal(s)	Feeding	Going outside	Putting to sleep	Playing	Obtaining babycare information
Independence without difficulty (3)	Goal	Decides on activities to complete in consideration of priority needs of baby and activity demands (e.g., schedule, length of activity) without compromising more urgent baby needs (*n* = 26)	x	x	x	x	x	x	x	x
		Monitors baby’s signs and interprets baby’s needs accordingly to formulate goals (e.g., hunger, fatigue) (*n* = 24)	x	x	x	x	x	x	x	
	Plan	Plans safe use of proper material or equipment (*n* = 19)	x	x	x	x	x	x	x	x
	Carry out	Accomplishes babycare safely, in environments with acceptable or no risks (*n* = 15)	x	x	x	x	x	x	x	x
		Carries out babycare activities while adequately interpreting baby’s signs and/or related babycare-related information (e.g., website content) (*n* = 13)	x	x		x	x		x	x
		Offers developmentally suitable options to baby (e.g., interactions, food, games) (*n* = 12)	x	x		x	x	x	x	
		Demonstrates knowledge of baby’s needs and routine in a timely fashion or, prior to assessment, inquires about such needs (*n* = 5)	x	x	x	x		x	x	
	Verify	Makes ongoing activity verifications and adjusts to activity demands as required (*n* = 15)	x	x	x	x	x	x		x
		Modifies plans and/or uses alternatives to meet the activity’s goal (*n* = 5)		x		x		x		x
With difficulty (2)	Goal	Has difficulty interpreting and/or anticipating baby’s needs, but manages without assistance (e.g., trial-and-error) (*n* = 8)		x	x	x		x	x	
		Initiates babycare after a certain delay, but manages to respond to baby’s needs in a reasonable amount of time (*n* = 5)		x	x	x		x	x	
	Plan	Only partially plans steps of activity, environmental adaptations, and/or related material which makes activity more difficult to carry out (e.g., limits interactions w/ baby, creates a longer course of action) (*n* = 17)	x	x	x	x	x	x	x	x
	Carry out	Has difficulty transporting, manipulating, finding, choosing and/or using items (e.g., clothing, equipment), but pursues activity safely (*n* = 14)	x	x	x	x				
		Has difficulty holding, transferring baby and/or positioning oneself with baby, but assumes it safely overall, with or without the use of a mobility aid or equipment (*n* = 10)	x	x		x		x	x	
		Has few responsive interactions w/ baby while the latter is awake (*n* = 4)	x	x		x		x	x	
		Demonstrates sup-optimal babycare knowledge, but does not compromise safety (e.g., recommended use of equipment) (*n* = 4)			x	x		x		
	Verify	Has difficulty completing, adjusting, or correcting actions to baby’s behaviors or needs, but does not compromise safety (*n* = 10)		x		x	x	x	x	
		Omits expected step(s) during babycare which reduces overall quality of babycare activity (*n* = 9)	x	x		x	x			
		Omits checking on or monitoring baby or babycare, but does not compromise baby’s safety (*n* = 8)			x	x		x	x	
Verbal and/or physical assistance (1v, 1p and/or 1vp)	Goal	Needs verbal assistance to initiate babycare, and/or prioritize which babycare to initiate, at proper time (*n* = 7)	x	x	x	x		x	x	
		Needs verbal assistance to interpret baby’s needs (e.g., hunger, discomfort) or gather additional babycare-related information (*n* = 7)		x	x	x	x	x	x	x
	Plan	Needs verbal assistance to safely plan babycare, including baby transfers (*n* = 11)	x	x	x	x	x	x	x	
		Needs verbal assistance to safely use, identify and/or choose the most appropriate alternative (e.g., material or equipment or environment for babycare) (*n* = 5)	x	x	x	x			x	
	Carry out	Needs punctual physical assistance to safely hold and/or transfer baby during babycare, with or without the use of equipment (*n* = 13)	x	x	x	x	x	x	x	
		Needs verbal assistance to maintain baby’s safety and/or comfort during babycare (e.g., positioning) (*n* = 11)		x	x	x	x	x	x	
	Verify	Needs verbal assistance to determine if babycare satisfies initial goal and whether it should be continued or resumed (*n* = 8)	x	x		x		x	x	
		Needs verbal assistance to account for encountered babycare difficulties or risks (*n* = 4)					x	x	x	
Dependence (0)	Goal	Perseveres with an activity that no longer meets baby’s changed needs and must be reoriented (*n* = 2)				x			x	
	Plan	Needs an important amount of verbal assistance to plan babycare (*n* = 8)	x	x	x	x		x		x
		Does not account for potential risks in the chosen environment for babycare (*n* = 3)	x	x		x		x	x	
		Initiates and/or plans babycare without respecting existing parent–baby safety recommendations (*n* = 2)		x	x	x		x		
	Carry out	Needs abundant verbal and/or physical assistance to execute many steps of babycare safely (e.g., problem solve) (*n* = 12)	x	x	x	x	x		x	x
		Needs to be stopped during activity to prevent evident safety risks (e.g., risks of falls or injuries, expired food) (*n* = 8)	x		x	x	x			
		Does not request assistance and puts baby at risk (e.g., by distancing oneself, during transfers) (*n* = 4)	x	x		x		x		
		Does not respect general hygiene, preparation and/or conservation measures during babycare (*n* = 4)	x	x	x	x				
	Verify	Does not resume, correct, or complete course of actions in a reasonable amount of time, despite unsatisfactory results, baby’s signs and/or assistance offered (*n* = 10)	x	x	x	x		x	x	x

*Note.* For each activity operation, categories are presented in decreasing prevalence of parents who demonstrated the corresponding behaviors or assistance needs. For each category, the corresponding sample of parents is reported in parentheses. The X marks indicate that the behavior or assistance need was demonstrated by at least one parent of the corresponding sample in the related babycare activity. ADL = Activities of Daily Living.

In sum, more than two thirds of all reported categories of salient parent behaviors or assistance needs were observed in at least half of the babycare activities. Interestingly, parents with the highest independence scores (i.e., scores of 3) manifested an array of flexible behaviors where interpretation, knowledge, and verification of baby’s needs were at the forefront, all the while taking into account the babycare activities’ intrinsic demands, proper use of material or equipment, and specific attention to safety within their home environments. As expected, the most frequently observed babycare activities had the most observed difficulties or assistance needs (i.e., scores of 2, 1, and 0). For those, observed throughout all babycare activities, the most prevalent categories of behaviors or assistance needs involved parents’ babycare planning, such as planning activity steps, environment, material/equipment, and/or baby transfers. Six other frequent assistance categories (i.e., scores of 1 and 0) involved punctual physical assistance, assistance with interpreting baby’s needs, carrying out many babycare steps safely, completing a specific course of action, initiating or prioritizing babycare at the proper time, and maintaining the baby’s safety or comfort, as observed throughout a majority of babycare activities. Finally, most assistance categories (i.e., scores of 1) involved verbal assistance rather than physical assistance, highlighting the cognitive demands associated with parenting.

## Discussion

The purpose of this qualitative observational study was to describe the assistance needs of parents with physical disabilities and cognitive impairments during common babycare activities carried out in their own homes. To our knowledge, this is the first study to conduct babycare observations in naturalistic settings with such a large sample of parents with a variety of physical disabilities. Through a collaboration with a unique clinic offering specialized rehabilitation services to parents with physical disabilities, most parents were provided with personalized adaptive babycare equipment to optimize their ability to carry out babycare activities with minimal physical assistance, and then observed as they completed a series of babycare activities in situ with their baby to document remaining assistance needs. During the study, assistance was only provided by therapists when safety risks or breakdowns in performance occurred, largely related to cognitive impairments which could not be compensated by the clinic’s adaptive equipment.

Overall, our findings showed that over 50% of our participants were dependent to bathe their babies, particularly for the planning and carrying out operations. About 40% were dependent to obtain information necessary for the care of their child, particularly due to high assistance needs to carry out the task and verify or interpret that they had obtained the required information. At least 25% of our sample had difficulties or required assistance (verbal and/or physical) at some point during the six other parenting activities assessed. Verbal assistance was required for large proportions of the sample to prioritize baby care at the proper time (i.e., formulate a goal), safely plan babycare (including transfers), safely use adaptive babycare equipment, and maintain the baby’s safety (i.e., carry out a task). Punctual physical assistance was required for a large proportion of our sample to safely hold and/or transfer baby during babycare, with or without the use of adaptive equipment.

Previous studies on parents with physical disabilities have noted the need for physical assistance or accessible accommodations in carrying out babycare activities such as bathing, sleeping, and breastfeeding which involve bending, lifting, and positioning (Pituch et al., 2020; Powell et al., 2019). Other studies have accounted for environments’ lack of accessibility (Kaiser et al., 2012), availability of adaptive equipment, or providers’ disability-related knowledge (National Council on Disability, 2012) which may all compromise parents’ participation in child care (Powell et al., 2019). Some research has highlighted the general difficulties associated with meal preparation or finding adapted babycare information due respectively to costs, scarce sources, or uninformed providers (Lipson & Rogers, 2000; Powell et al., 2019). In addition, playing has been previously described as one of the most challenging activities due to the physical demands it entails (Pituch et al., 2020). Indeed, in a recent survey, “more assistance from others” was the most frequent answer of mothers with physical disabilities to optimize physical care, comforting, playing, setting limits, and outings with young children (Jacob et al., 2017). However, the need for specific forms of assistance and at different steps of babycare activities (e.g., formulating a goal, planning, or verifying goal attainment), such as verbal assistance (e.g., prompts, reminders), has been largely unacknowledged until this study. Likewise, assistance with babycare planning was until now rarely accounted for, despite the added steps of using certain necessary alternatives (e.g., equipment) with concurrent consideration of both the parent’s own security and abilities, and the baby’s needs.

This study was guided by a strong will to support parenting by better understanding the assistance needs of parents with physical disabilities, all the while acknowledging their primary caregiver role in a safe assessment context. Such an approach represents a major paradigm shift. Many studies indicate that parents with disabilities are too often considered as individual receivers of care rather than caregivers (Campion, 1995) or, conversely, that their parenting activities are accomplished for them rather than with them (Martinsen et al., 2012). However, maintaining a primary caregiver status is important for many parents needing assistance (Kaiser et al., 2012). Our study highlights the urgent need to develop support systems and interventions that facilitate easier and safer babycare at home. While all parents and mostly first-time parents may have practical caregiving needs in the early postnatal period (Entsieh & Hallström, 2016), our study portrays specific babycare needs that could be met should timely assistance from coparents and/or community providers (Wint et al., 2016) be made available. Although hands-on in-home supports appear to be lacking for parents with physical disabilities (Malacrida, 2009), our study findings open the way to exploring countless possibilities that could mitigate some parents’ lived difficulties and in-home risks in terms of parenting knowledge and skills (e.g., adapted parenting strategies), babycare activities [e.g., sharing parenting responsibilities, routines and/or steps, adapting activities, Powell et al., 2019; Kaiser et al., 2012], service provision (e.g., verbal and/or physical assistance, cognitive-based training), adaptations, or technological solutions (National Council on Disability, 2012), all of which should be wisely chosen by informed stakeholders. Finally, future studies and practice should make sure that parents remain central in babycare while receiving the necessary assistance (Jacob et al., 2017).

Although formal, in-home, and observation-based assessments are currently rarely performed (Lampe et al., 2019; National Council on Disability, 2012), our study suggests the utmost importance of conducting individualized, case-by-case babycare assessments within the lived environments (Major et al., 2018) to avoid incorrect assumptions or overgeneralizations of parents’ assistance needs with their potentially dramatic consequences [e.g., termination of parental rights, Campion, 1995]. Our study results must also be interpreted cautiously, considering that parents’ captured performances are situational and shaped by their (parenting) experiences, their individual and baby’s functioning, and their environments, all of which are constantly evolving and could be potentially optimized with better supports in place.

### Limitations and Future Directions

This study had certain limitations. First, due to a current lack of validated performance-based parent measures (Pastor-Bédard et al., 2023), our home-based observations used an adapted version of the *ADL Profile* for which psychometric studies have not yet been completed. Thus, steps were taken to limit inter-rater variability between the dyads (e.g., training, only two researcher OTs throughout the study). Second, with our assessments being announced and recorded, a social desirability bias is possible. Also, despite our representation efforts, only a third of our sample were fathers. Finally, although we had a fair sample size in this study, it is possible that we did not capture all babycare needs, especially for parents who accomplish other activities at different periods (e.g., night), care for older babies, or do not have access to (adaptive) parenting equipment. Future studies should investigate individual differences in terms of assistance needs within parents’ environments.

## Conclusion

This qualitative study described in-home parenting assistance needs. It is one of the first community occupational therapy-based studies involving parent–baby observations using an analytic framework focused on examining the impact of possible cognitive impairments, particularly EF, on babycare activities. This study provided a detailed description of parents’ strengths, difficulties, and assistance needs, expanding our knowledge of early parenting with physical disabilities. Community providers need to work with parents with physical disabilities to provide thought-out solutions to their babycare needs.
